# Real-time estimations of blood glucose concentrations from sweat measurements using the local density random walk model

**DOI:** 10.1007/s11517-025-03393-z

**Published:** 2025-06-07

**Authors:** Xiaoyu Yin, Elisabetta Peri, Eduard Pelssers, Jaap den Toonder, Massimo Mischi

**Affiliations:** 1 Department of Electrical Engineering, Eindhoven University of Technology, Eindhoven , Noord-Brabant Netherlands; 2 Department of Mechanical Engineering, Eindhoven University of Technology, Eindhoven , Noord-Brabant Netherlands

**Keywords:** Sweat sensing, Diabetes, Patient monitoring, Pharmacokinetic modeling

## Abstract

Sweat provides a non-invasive alternative to blood draws, enabling glucose-concentration monitoring for both healthy individuals and diabetic patients. In our previous work, we demonstrated a strategy that accurately estimates blood glucose concentrations from sweat measurements. However, this method involves time-consuming simulations using a biophysical model, limiting its application to offline use. The goal of this study is to propose an approach that increases computational efficiency, thereby facilitating real-time estimation of blood glucose concentrations using sweat-sensing technology. To this end, we propose replacing the original biophysical model with the Local Density Random Walk (LDRW) model. This is justified because both models describe the pharmacokinetics of glucose transport through a convective-diffusion process. The performance of the LDRW model and the original biophysical model are compared in terms of estimation accuracy, computational efficiency, and model complexity, using seven datasets from the literature. The estimation of blood glucose concentrations using the LDRW model closely approximates that of the original model, with a root mean square difference of just 0.04 mmol/L between the two models' estimates. Remarkably, the LDRW model significantly reduces the average computational time to 2.6 s per data point, representing only 0.7% of the time required by the original method. Furthermore, the LDRW model demonstrates a smaller corrected Akaike Information Criterion value than the original method, indicating an improved balance between goodness of fit and model complexity. The proposed novel approach paves the way for the clinical adoption of sweat-sensing technology for non-invasive, real-time monitoring of diabetes.

**Graphical Abstract:**

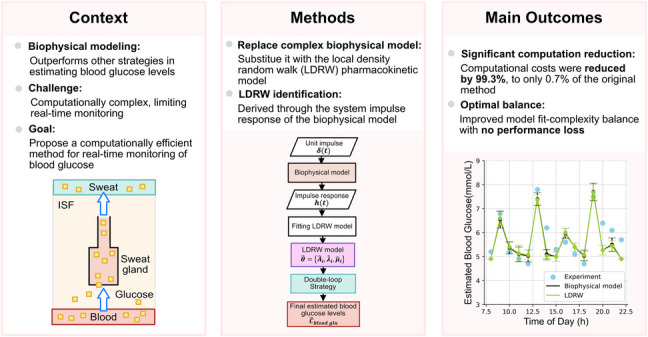

## Introduction

Diabetes is a metabolic disorder characterized by elevated blood glucose concentrations due to insufficient production or ineffective use of insulin [1]. In recent years, this disease has gained increasing attention due to its prevalence across populations. In 2021, approximately 537 million people were living with diabetes, representing 10.5% of the overall population worldwide [2]. The mortality rate associated with diabetes and its complications was as high as 6.7 million deaths in 2021 [2]. Diabetes-related vascular complications such as cardiovascular disease, diabetic kidney disease, and diabetic retinopathy are major factors contributing to illness and death [3–5]. These complications not only reduce the overall quality of life and lead to higher mortality rates but also result in substantial economic burdens [4, 6], reaching 966 billion U.S. dollars globally in 2021 [2].

To prevent and mitigate diabetic complications, early diagnosis and timely medical intervention are pivotal [1, 7, 8]. A current challenge in diabetes management involves customizing medication doses and timing to meet the individual patient needs [9]. Specifically, medication doses should be adjusted in response to an individual’s current blood glucose concentrations. Real-time monitoring of blood glucose is essential, as it allows precise and timely adjustments of medication doses. However, common methods for monitoring blood glucose concentrations are typically invasive. The fingerstick test is the most prevalent method and it requires pricking the fingertip to obtain a small blood sample [10]. Another sampling concept employs microneedles for transdermal sampling of the interstitial fluid (ISF) [11]. These invasive techniques may cause discomfort and potential harm, and are prone to occlusions and challenges in obtaining adequate sample volume, limiting the frequency of sampling and potentially impacting patient compliance [12, 13]. The limitations of existing methods for blood glucose monitoring highlight the need for real-time, non-invasive alternative methods.

To enable non-invasive real-time monitoring of blood glucose concentrations, sweat-sensing technology emerges as a valuable option, offering an alternative to traditional blood and ISF sampling. However, research studying the relationship between blood and sweat glucose concentrations is still very limited. The study by Moyer et al. achieved only a moderate determination coefficient (${R}^{2}$) of 0.69 from a total of 115 experimental samples, utilizing linear regression analysis [14]. Klous and colleagues reported a correlation coefficient of 0.73 from a total of 48 data points using linear regression analysis [15]. Our previous research proposed a biophysical model to characterize glucose transport across various compartments as a convective-diffusion process, simulating the pharmacokinetic transport of glucose from blood capillaries to sweat excretion. Building on this model, we introduced a novel double-loop optimization strategy. This approach effectively solves the inverse problem, allowing for the estimation of blood glucose concentrations based on measured sweat glucose concentrations [16]. This double-loop strategy consists of two intertwined optimization loops, both employing an optimizer to reduce the error between measured and estimated sweat glucose concentrations. The first loop is dedicated to optimizing blood glucose concentrations, which are subsequently used as inputs for the biophysical model. The second loop aims at refining the parameters of the biophysical model in a personalized way. This approach achieved a high Pearson correlation coefficient ($R$) of 0.98 using 108 experimental data points for estimating blood glucose from sweat glucose measurements.

While the double-loop optimization strategy is currently the most accurate method for estimating blood glucose concentrations via sweat-sensing technology, the substantial computational time requirements hamper its application for real-time monitoring. This strategy involves time-intensive simulations using a biophysical model implemented in COMSOL Multiphysics, requiring complete simulations for each iteration within both optimization loops. With multiple iterations typically necessary, the computational times generally range from 5 to 10 min per data point. Consequently, this duration far exceeds the thresholds for real-time estimation, necessitating more efficient computing. To address this challenge, our objective is to reduce the computational time of this strategy without compromising its accuracy. A simplified mathematical model, offering fewer parameters and reduced complexity, could be advantageous in improving computational efficiency through model reduction, providing a more feasible alternative to the original, complex biophysical model.

The biophysical model we employ to describe the convective-diffusion process of glucose transport is inherently complex. However, the Local Density Random Walk model (LDRW) can provide a simpler alternative for modeling convection–diffusion transport processes in pharmacokinetics. This model, originally developed to interpret and fit Indicator-Dilution Curves (IDC) in thermo- or dye-dilution studies, has been adapted for contrast-enhanced imaging applications [17–19]. The LDRW model operates by analyzing the probability density function of an indicator detected at a specific distance downstream following its upstream injection into an infinitely long tube. This transport is modeled as a convective-diffusion process, which describes the spatial and temporal evolution of the indicator concentration, solved under given boundary conditions. The LDRW model accommodates multiple passages of the indicator [17], providing an accurate representation of biomarker transport processes and making it well-suited for our study. Unlike the biophysical model, which uses 18 parameters and 10 equations as characterized in [16], the LDRW model simplifies the description of the biomarker transport process, e.g. for indicator molecules or particles, using a single analytical function with 3 parameters only. This model reduction, achieved through the LDRW model, is expected to accelerate the model identification by parameter estimation and enhance its reliability.

This study aims at developing a method for real-time estimation of blood glucose concentrations based on sweat glucose measurements. Building upon our previously introduced double-loop optimization strategy in [16], this study proposes replacing the original biophysical model with the LDRW model to enhance computational efficiency. To assess the effectiveness of our approach, we compare the results from this study with those of our previous work, in terms of estimation accuracy as well as computational time. Additionally, we conducted two separate sensitivity analyses: the first to evaluate the sensitivity of the glucose concentration estimates to the model parameters, and the second to explore the interlink between the parameters of the LDRW and the original biophysical model, thus investigating the physiological relevance of the LDRW model parameters.

## Methods

### Biophysical model and its impulse response

**Fig. 1:**
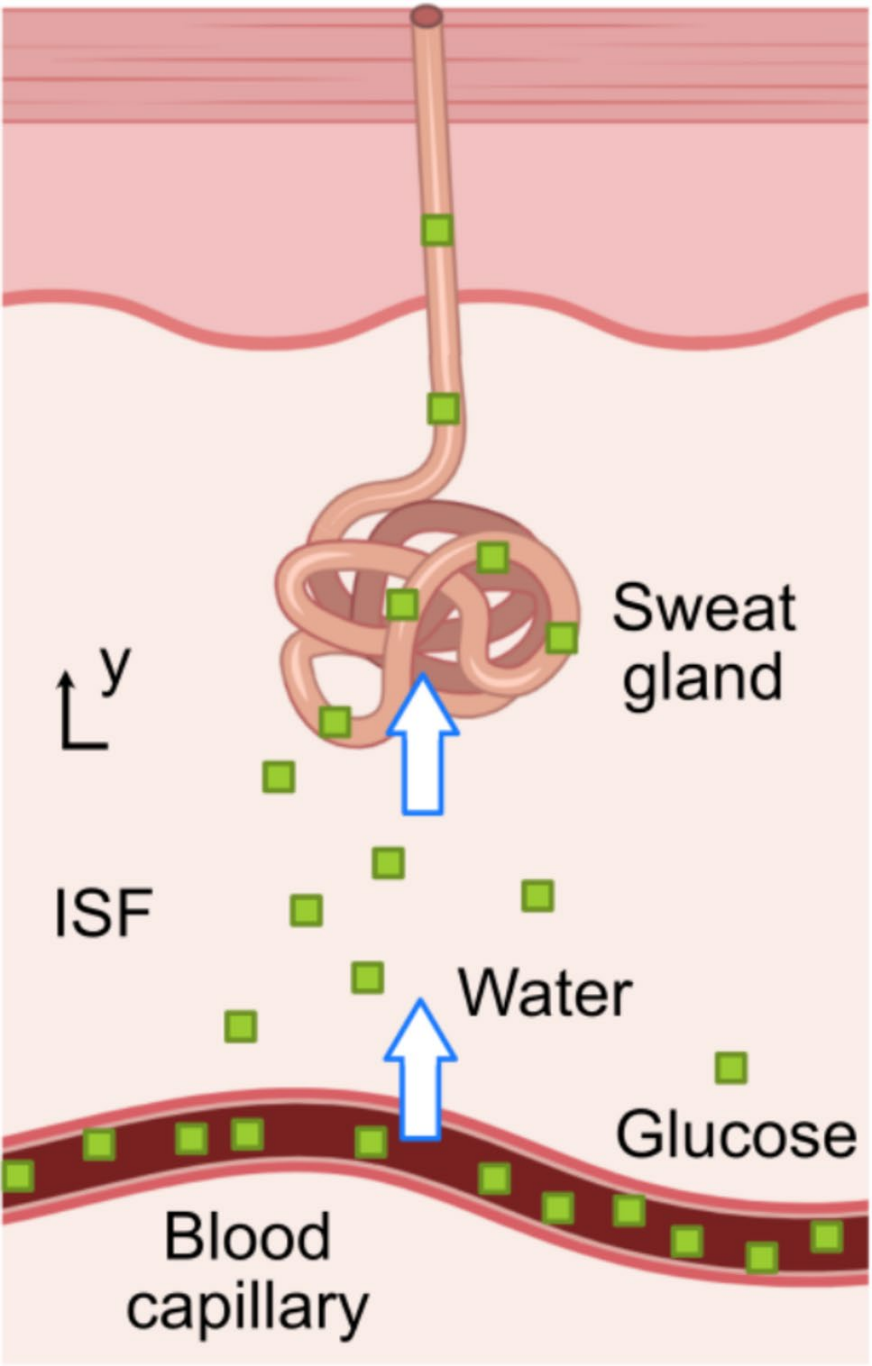
Schematic illustration of the biophysical model for glucose transport

In our previous work, we proposed a compartmental biophysical model using COMSOL Multiphysics to simulate the transport of glucose from blood capillaries to sweat along a sweat gland [16], as illustrated in Fig. 1.

In this section, we summarize the model working principle and we introduce the 18 model parameters used. The full model details are provided elsewhere [16]. The glucose transport is described considering three main steps. Firstly, glucose in the blood capillary compartment is transported to the interstitial fluid (ISF) compartment. This glucose flow, denoted as ${J}_{source}$ in $mol {s}^{-1}$, is driven by the difference in glucose concentrations between the blood capillary ${C}_{p}$ and the ISF compartment ${C}_{ISF}$, both in $mol {m}^{-3}$, given as

1 $${J}_{source}={k}_{DE}({C}_{p}-{C}_{ISF}){V}_{p},$$

where ${V}_{p}$ is the blood capillary compartment volume related to a single sweat gland in ${m}^{3}$ [20], and ${k}_{DE}$ represents the glucose clearance constant in the dermis in ${s}^{-1}$[21].

At the same time, water moves from the blood capillary compartment to the ISF compartment due to hydrostatic pressure gradients between these two compartments. The water flow rate, ${Q}_{water}$, is governed by the Starling equation as

2 $${Q}_{water}={L}_{p,c}{A}_{c}\left({P}_{c}-{P}_{ISF}\right),$$

where ${P}_{c}$ represents the hydrostatic pressure in the capillaries in $mmHg$[22], ${P}_{ISF}$ represents the hydrostatic pressure in the ISF in $mmHg$ [22], ${L}_{p,c}$ is the hydraulic conductivity in the capillaries in $m{s}^{-1}{mmHg}^{-1}$[23], and ${A}_{c}$ is the blood capillary compartment area related to a single sweat gland in ${m}^{2}$ [20].

The water flow velocity in the ISF compartment, ${u}_{ISF}$ ($m{s}^{-1}$), is obtained by dividing the ${Q}_{water}$ by the ISF compartment's cross-sectional area ${A}_{ISF}$ (${m}^{2}$),

3 $${u}_{ISF}=\frac{{Q}_{water}}{{A}_{ISF}}.$$

After glucose enters the ISF compartment, it is subject to diffusion and convection processes. During these processes, a fraction of the glucose flow, denoted as ${J}_{source}$, is absorbed by cells at a rate ${r}_{uptake}$ ($mol{m}^{-3}{s}^{-1}$) [24], given as

4 $$\frac{\partial {C}_{ISF}}{\partial t}=\frac{{J}_{source}}{{V}_{ISF}}-{r}_{uptake}+{D}_{ISF}\frac{{\partial }^{2}{C}_{ISF}}{\partial {y}_{ISF}^{2}}-{u}_{ISF}\frac{\partial {C}_{ISF}}{\partial {y}_{ISF}},$$

with ${V}_{ISF}$ the ISF compartment's effective volume in ${m}^{3}$ [20], ${D}_{ISF}$ is the glucose diffusion coefficient in the ISF in ${m}^{2}{s}^{-1}$ [25], ${y}_{ISF}$ is the distance covered by glucose from its entry point into the ISF compartment in $m$. Notice that the glucose diffusion coefficient, ${D}_{ISF}$, is considered spatially constant over the ISF in Eq. (2).

Due to the concentration gradient existing between the ISF compartment and the sweat gland compartment, glucose in the ISF subsequently transitions into the sweat gland. Diffusion primarily governs the passage of glucose across the sweat gland wall, which can be characterized using Fick's first law as.

5 $${J}_{ISF-sg}={D}_{sg,wall}\frac{\partial \left({C}_{ISF}-{C}_{sg}\right)}{\partial {h}_{sg}},$$

where ${J}_{ISF-sg}$ is the glucose flux entering the sweat gland in $mol {s}^{-1}$, ${D}_{sg,wall}$ represents the glucose diffusion coefficient in the sweat gland wall (${m}^{2}{s}^{-1}$) [26], ${h}_{sg}$ is the sweat gland wall thickness in $m$ [27], and ${C}_{sg}$ is the concentration of glucose in the sweat gland in ${mol m}^{-3}$.

Water also flows into the sweat gland compartment at a flow rate ${Q}_{water,sg}$ in ${m}^{3}{s}^{-1}$, and subsequently travels along the sweat gland toward the skin surface. This process is governed by a pressure difference $\Delta P$ between the ISF and the skin surface through the sweat gland in $mmHg$ [28] and can be given using Darcy's law as

6 $${Q}_{water}=\frac{\Delta P}{R},$$

where $R$ represents the sweat gland's hydraulic resistance in $Pa\bullet s {m}^{-3}$ [27], which can be given as

7 $$R=\frac{128\eta L}{\pi {d}^{4}},$$

where $\eta$ is the water viscosity in $Pa\bullet s$ [29], $L$ is the sweat gland length in $m$ [30], and $d$ is the sweat gland's luminal diameter in $m$ [31].

The water flow velocity in the sweat gland, ${u}_{sg}$ ($m{s}^{-1}$), can be given as

8 $${u}_{sg}=\frac{{Q}_{water,sg}{u}_{sweat,n}}{{A}_{sg}},$$

where ${Q}_{water,sg}$ is the ratio of the water flow rate, ${A}_{sg}$ is the sweat gland's luminal area, and ${u}_{sweat,n}$ is the experimental sweat velocity normalized by the passive sweat velocity, which is $3\times {10}^{-4} m{ s}^{-1}$[32]. ${u}_{sweat,n}$ servers as a correction factor to adjust ${u}_{sg}$ for differences between passive sweating and experimental conditions, such as sauna, high-intensity exercise, and stimulated sweating.

Lastly, the glucose is transported through the sweat gland and ultimately reaches the human skin surface. This process can also be described as a convective-diffusion process, similar to Eq. (4), with ${C}_{sg,dil}$ denoting the glucose concentration in the sweat gland compartment in $mol {m}^{-3}$,

9 $$\frac{\partial {C}_{sg,dil}}{\partial t}={D}_{sw}\frac{{\partial }^{2}{C}_{sg,dil}}{\partial {y}_{sg}^{2}}-{u}_{sg}\frac{\partial {C}_{sg,dil}}{\partial {y}_{sg}},$$

where ${D}_{sw}$ is the spatially-invariant glucose diffusion coefficient in water in ${m}^{2}{s}^{-1}$ [26].

The dilution effect of water influxes on glucose concentration at different sweat rates is given as

10 $${C}_{sg,dil}=\frac{{C}_{sg}}{1+{K}_{w/g}{u}_{sg,n}},$$

where ${K}_{w/g}$ represents the ratio between the volumetric flow rate of water and glucose [33], and ${u}_{sg,n}$ is the normalized water velocity in the sweat gland (${u}_{sg}$) relative to the passive sweat velocity.

For a more detailed description of the biophysical model, please refer to our previous work [16]. All model parameters are listed in Table 1.

**Table 1:** Parameter of the biophysical model

Parameter	Unit	Value	Ref
Capillary hydrostatic pressure: P_c_	mmHg	30	[22]
Capillary hydraulic conductivity: L_p,c_	m s^−1^ mmHg^−1^	6.5 × 10^–10^	[23]
Clearance constant of glucose: k_DE_	s^−1^	6.51 × 10^–4^	[21]
Effective volume of capillary: V_p_	m^3^	3.02 × 10^–13^	[20]
Effective volume of ISF: V_ISF_	m^3^	6.0 × 10^–13^	[20]
Hydrostatic pressure in ISF: P_ISF_	mmHg	−3	[22]
Glucose diffusion coefficient for sweat gland wall: D_sg,wall_	m^2^ s^−1^	6.46 × 10^–10^	[26]
Glucose diffusion coefficient in ISF: D_ISF_	m^2^ s^−1^	2.64 × 10^–10^	[25]
Glucose diffusion coefficient in water: D_sw_	m^2^ s^−1^	6.7 × 10^–10^	[26]
Luminal diameter of sweat gland: d	m	5 × 10^–6^	[31]
ISF cross-sectional area: A_ISF_	m^2^	2.2 × 10^–8^	[20]
Pressure difference across sweat gland: ΔP	mmHg	10	[28]
Ratio of volumetric flow rate of water to glucose: K_w/g_	-	12	[33]
Sweat gland length: L	m	4 × 10^–3^	[30]
Sweat gland wall thickness: h_sg_	m	5 × 10^–5^	[27]
Surface area of capillary: A_c_	m^2^	1.5 × 10^–8^	[20]
Uptake rate of glucose: r_uptake_	mol m^−3^ s^−1^	2.78 × 10^–2^	[24]
Water viscosity: η	Pa·s	1 × 10^–3^	[29]

The mechanism described by the biophysical model can be approximated as linear and time-invariant (LTI) within a short time frame. This approximation allows us to characterize the system through its impulse response, which in LTI systems describes how the system's output changes over time in response to an impulse. This response not only captures the characteristics of the system but also enables prediction of system's response to any input signal. Specifically, the system's output can be determined by the convolution of its input with the impulse response [34].

To investigate the impulse response of the biophysical model, we input a Dirac delta function, denoted as $\delta (t)$, into the model [35, 36]. The response of the biophysical model to this input is termed its impulse response, which is represented by $h(t)$.

### LDRW model and model identification

**Fig. 2:**
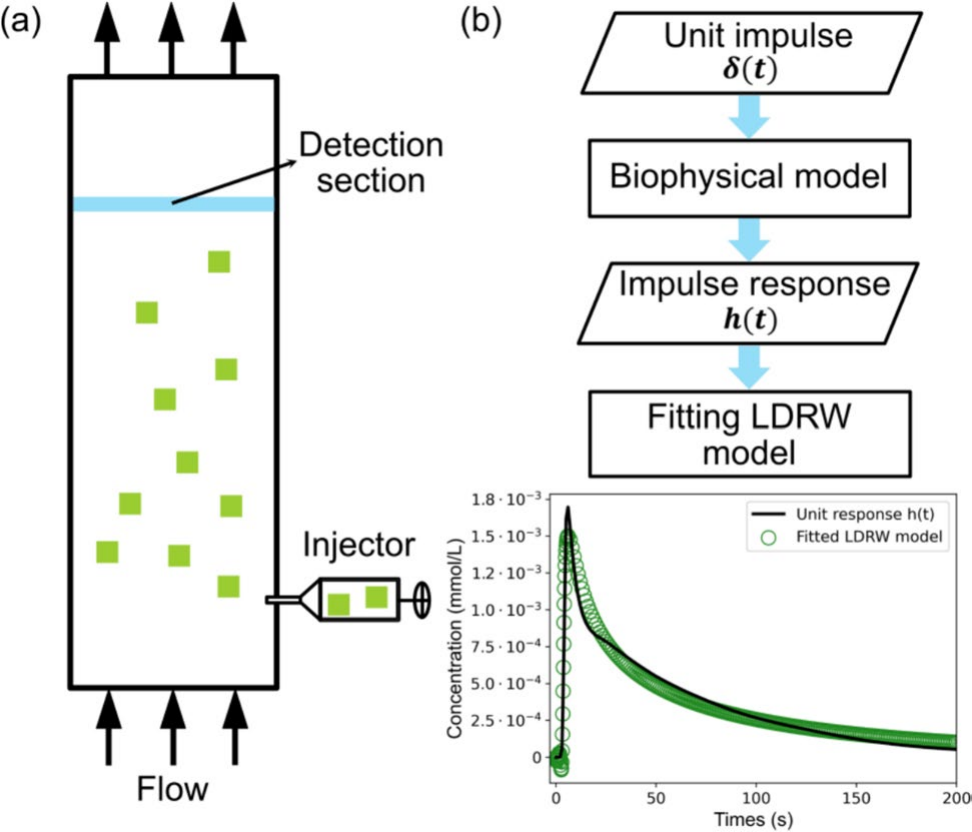
(**a**) Schematic illustration of the LDRW experimental model. (**b**) Flowchart for Identifying the Initial Parameters of the LDRW Model Using the Impulse Response of the Biophysical Model

As mentioned in the previous section, our biophysical model employs 18 parameters to characterize glucose transport, primarily as a process dominated by convection and diffusion, as described in Eqs. (4) and (9). The convective-diffusion process also forms the basis of extensive research focused on modeling the time evolution of the concentration of an indicator, represented by its IDC, following its rapid injection (see Fig. 2(a)) [19]. In general, a diffusion-convection process is described as

11 $$\frac{\partial {c}_{i}\left(z,t\right)}{\partial t}=\frac{\partial }{\partial z}\left({D}_{i}\frac{\partial {c}_{i}\left(z,t\right)}{\partial z}\right)-v\frac{\partial {c}_{i}\left(z,t\right)}{\partial z},$$

where ${c}_{i}(z,t)$ is the indicator concentration in $mol {m}^{-3}$, ${D}_{i}$ is the diffusion coefficient of the indicator in ${m}^{2}{s}^{-1}$, $v$ is the fluid flow rate in $m {s}^{-1}$, $z$ is the coordinate along the longitudinal dimension in $m$.

The LDRW model provides a solution to the diffusion-convection Eq. (11) under specific boundary conditions [37]. The fast injection hypothesis is represented by the initial condition as

12 $${c}_{i}\left(z,0\right)=\frac{m}{S}\delta \left(z\right),$$

where $m$ is the solute dose in $mol$ and $S$ is the cross-sectional area of the tube in ${m}^{2}$. The boundary condition ensuring mass conservation during transport is expressed as

13 $${\int }_{0}^{\infty }{c}_{i}\left(z,t\right)dz=\frac{m}{S}.$$

**Fig. 3:**
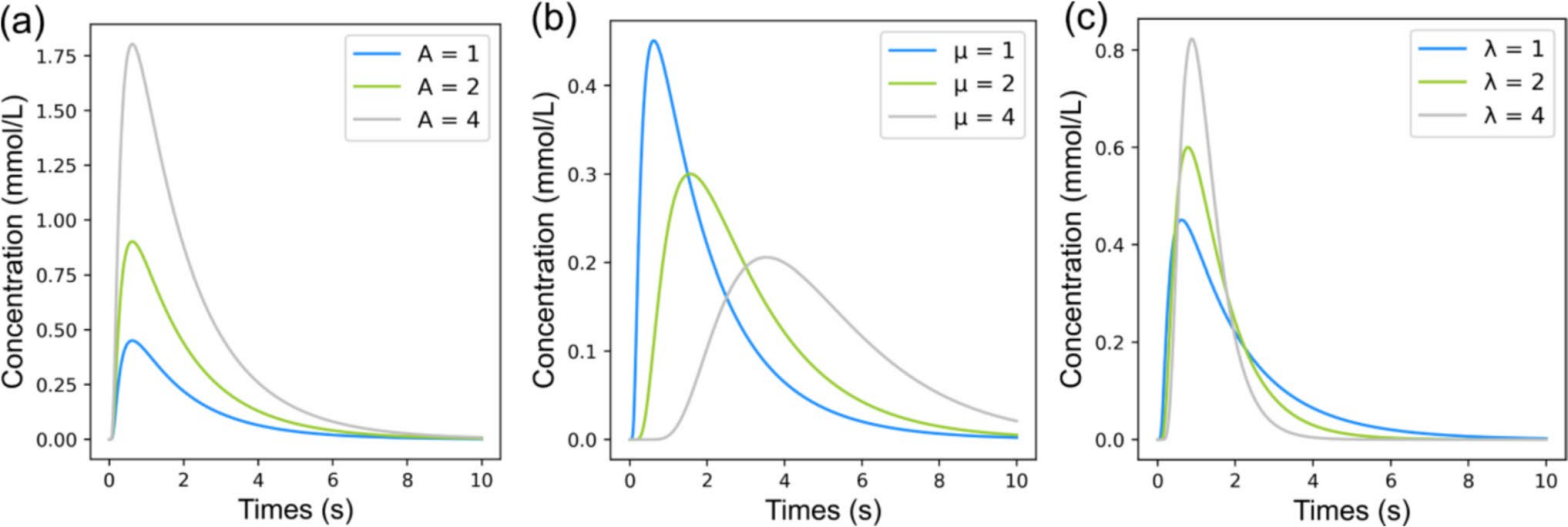
Impact of model parameters on the LDRW model response. (**a**) Effect of varying $A$ ($A=1, 2, 4$) with $\mu =1 s$ and $\lambda =1$. (**b**) Effect of varying $\mu$ ($\mu =1, 2, 4$) with $A=1 mol {s}^{-1}{m}^{-3}$ and $\lambda =1$. (**c**) Effect of varying $\lambda$ ($\lambda =1, 2, 4$) with $A=1 mol{ s}^{-1}{ m}^{-3}$ and $\mu =1 s$

Given these conditions, the concentration–time profile of the LDRW model at the detection section is given as:

14 $$C\left(t\right)=A{e}^{\lambda }\sqrt{\frac{\lambda }{2\pi \mu t}}{e}^{-\frac{\lambda }{2}\left(\frac{t}{\mu }+\frac{\mu }{t}\right)},$$

where $A$ in $mol s {m}^{-3}$ is the ratio of the injected dose of solute $m$ in $mol$ to the volumetric flow of the carrier fluid $Q$ in ${m}^{3}{s}^{-1}$ ($Q=vS$), $\mu$ is the mean transit time of the solute from the injection to the detection section in $s$, $\lambda$ is a dimensionless parameter that indicates the skewness of the curve and is equal to half of the Peclet number ($Pe/2$), which represents the ratio of convection to diffusion of a solute. These three parameters define the LDRW model. The impact of variations in $A$, $\mu$, and $\lambda$ on the LDRW model response is illustrated in Fig. 3.

The initial parameter values for the LDRW model, denoted as ${\theta }_{0}=\left\{{A}_{0}, {\lambda }_{0}, {\mu }_{0}\right\}$, were identified by fitting the impulse response, $h(t)$, derived from the biophysical model. This fitting was accomplished using a multiple linear regression (MLR) algorithm in the logarithmic domain, developed by our group [37], which has demonstrated optimal performance for LDRW fitting, as detailed in our prior works [17, 37]. The whole process is illustrated in Fig. 2(b).

### Inverse estimation by double-loop optimization

**Fig. 4:**
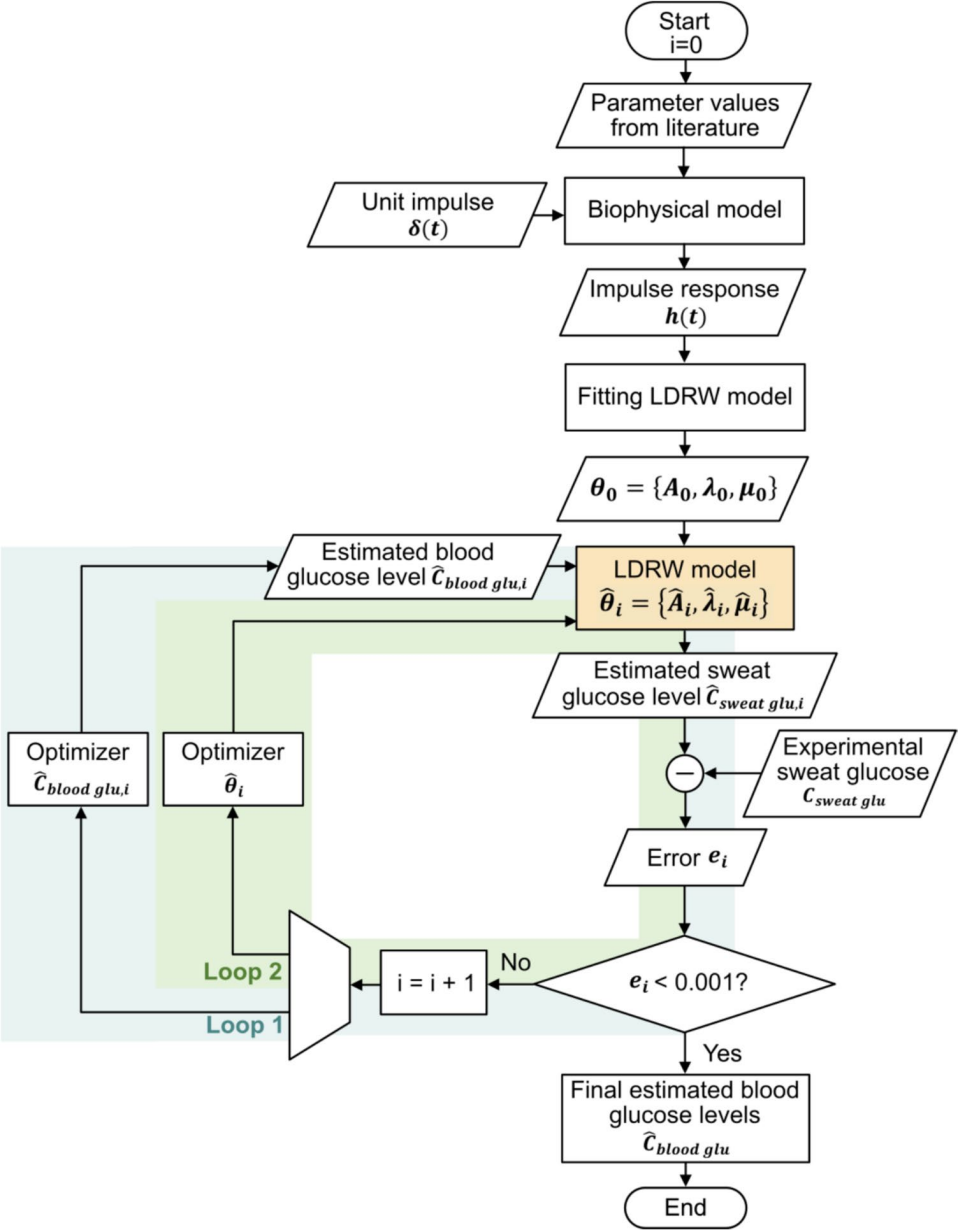
Flowchart of double-loop optimization strategy for inverse estimation of blood glucose concentrations based on sweat measurements. The optimization objective of loop 1 is to estimate the blood glucose concentration, and the optimization objective of loop 2 is to fine-tune the parameters of the LDRW model. The loops operate in an alternating manner, with one cycle involving loop 1 and the subsequent one involving loop 2

Inverse estimation is a method used to infer the input of a system based on its output. In our study, this involves estimating blood glucose concentrations from sweat measurements. This approach is particularly important for enabling non-invasive monitoring of blood glucose concentrations, given the unclear relationship between sweat and blood glucose concentrations. The overall strategy for inverse estimation using the LDRW model is outlined in Fig. 4 and is explained in detail below:

Initially, the complex biophysical model is used only once to characterize the system via its impulse response $h(t)$. $h(t)$ is obtained by applying $\delta (t)$ to the biophysical model, which is parameterized using values from the literature listed in Table 1. The initial parameters of the LDRW model, ${\theta }_{0}=\left\{{A}_{0}, {\lambda }_{0}, {\mu }_{0}\right\}$, are then identified by fitting $h(t)$ with the LDRW model expression. Subsequently, the double-loop optimization strategy focuses on refining the three parameters of the more computationally efficient LDRW model, rather than the 18 parameters of the complex biophysical model. In the double-loop strategy, the first loop optimizes the model input, specifically the estimated blood glucose concentration, starting with a typical initial value ${C}_{blood glu,0}$ of $5.5 mmol {L}^{-1}$, based on average values in healthy individuals [38]. The second loop focuses on fine-tuning the model parameters, $\theta$, to tailor the LDRW model in a personalized way. These loops are intertwined, with one iteration for input optimization followed by one iteration for parameter optimization. This iterative process refines the estimated blood glucose concentrations and the model parameter values. For each iteration $i$, the error ${e}_{i}$, defined as the mean square error between the estimated sweat glucose ${\widehat{C}}_{sweat glu,i}$ and the experimental sweat glucose ${C}_{sweat glu,i}$, is calculated. The optimization process continues until ${e}_{i}$ falls below 0.001. To mitigate the risk of convergence to local optima, both loops in the algorithm are executed only once per optimization iteration. For a more comprehensive explanation of the double-loop optimization strategy, readers can refer to our previous work [16].

In our implementation, MLR in the logarithmic domain provides a fast and robust initialization of the LDRW model parameters. This linear approach avoids the convergence issues faced by traditional nonlinear methods when dealing with noisy data [37]. Yet, applying a log transformation, the noise characteristics change too, making a least-squares optimization sub-optimal. Therefore, following this initialization step, the double-loop strategy employs a sparse nonlinear optimizer (SNOPT) [39] without log-transformation in both optimization loops. SNOPT’s sequential quadratic programming framework efficiently handles large-scale, sparse problems, making it particularly suitable for multi-parameter estimations. Specifically, it is ideal for iteratively refining the biophysical model's 18 parameters and the estimated blood glucose concentration ${\widehat{C}}_{blood glu}$. Additionally, its line search approach ensures that the algorithm dynamically adjusts the step length to converge to an optimal solution even with suboptimal initialization.

### Data sources

The described strategy is tested on seven sets of experimental data from the literature [14, 15, 40–43], as detailed in Table 2. The experiments were conducted under different conditions, including meal intake, fasting, and glucose ingestion, to induce variations in blood glucose concentrations. The participants included 18 healthy individuals and 2 diabetic patients, resulting in a total of 108 paired blood and sweat glucose measurements. Such diversity in experimental conditions and subject profiles is critical for validating the robustness and generalizability of the proposed estimation strategy across different physiological states. Specifically, datasets corresponding to Experiments 1–6 (as listed in Table 2) are publicly available through the following references: [14, 40–43]. The original data and experimental procedures can be accessed in the respective publications. The dataset corresponding to Experiment 7 was acquired through collaboration with Vrije Universiteit Amsterdam, as detailed in [15]. This study was approved by the Medical Ethics Committee of Erasmus MC Rotterdam (MEC-2019–0202) and conducted in accordance with the revised Declaration of Helsinki (2013).

**Table 2:** Experimental dataset of sweat and blood measurements

Data Sources	Participant health condition	Sweat glucose (Mean ± Std) [μmol L^−1^]	Blood glucose (Mean ± Std) [mmol L^−1^]	Measuring points number
Exp 1[40]	1 healthy	97.1 ± 13.6	5.6 ± 0.9	15
Exp 2[41]	2 healthy	37.5 ± 22.4	11.4 ± 5.7	19
Exp 3[14]	1 diabetic	178.3 ± 34.3	15.4 ± 2.1	6
Exp 4[42]	1 healthy	19.3 ± 12.1	5.0 ± 0.1	7
Exp 5[42]	1 healthy	42.4 ± 26.4	4.7 ± 0.6	5
Exp 6[43]	1 Healthy and 1 Diabetic	69.4 ± 36.3	6.4 ± 1.2	8
Exp 7[15]	12 healthy	12.8 ± 4.9	4.6 ± 0.6	48

### Model evaluation and performance metrics

The evaluation procedure of both the biophysical model and the LDRW model was organized into four aspects: estimation performance, computational efficiency, model complexity, and parameter sensitivity. The detailed assessment methods are described below.

#### Estimation performance

Firstly, we computed several performance metrics to evaluate and compare the estimation results of the biophysical model and the LDRW model against the corresponding experimental blood glucose values. These metrics included the Root Mean Square Error (RMSE), Mean Absolute Error (MAE), Root Mean Square Percentage Error (RMSPE), the Pearson correlation coefficient ($R$), and the coefficient of determination (${R}^{2}$) [44]. Secondly, to assess the agreement between the two models directly, we calculated the Root Mean Square Difference (RMSD) between their respective estimated blood glucose concentrations for each experimental data point. To evaluate whether the differences in estimation results between the two models were statistically significant, a paired two-tailed Wilcoxon signed-rank test was performed.

#### Computational efficiency

To evaluate the computational speed of the biophysical and LDRW models, the computational time for each model under identical testing conditions was recorded. Specifically, we documented the computational time per data point for the two models and calculated the ratio of the computational time required by the biophysical model to that of the LDRW model. A ratio exceeding 1 indicates that the biophysical model is more time-consuming, while a ratio less than 1 implies that it is faster. A paired two-tailed Wilcoxon signed-rank test was conducted on the computational time per data point for each model to assess whether the observed differences in computational efficiency was statistically significant.

#### Model complexity

To assess the goodness-of-fit and complexities of both models, the corrected Akaike Information Criterion (AICc) values were calculated. While the standard Akaike Information Criterion (AIC) is commonly used for model selection [45], the AICc is preferred in cases of limited sample sizes, as it minimizes the risk of overfitting [46]. In our study, we computed the AICc values by comparing the estimated blood glucose concentrations from the double-loop optimization strategy for both the LDRW and biophysical models against the experimental values. The AIC is formulated as

15 $$AIC=2k-2\text{ln}\left(L\right),$$

where $k$ is the number of estimated parameters in the model, and $L$ is the maximum value of the likelihood function for the model. Given the limited sample size $n$, the AICc was employed, which is given as

16 $$AICc=AIC+\frac{2k\left(k+1\right)}{n-k-1}.$$

In model selection, the model with lower AICc value is considered optimal, as it represents the better trade-off between goodness of fit and model complexity.

#### Sensitivity analysis

Two sensitivity analyses were conducted on the two models'parameters. The first analysis aimed to evaluate how variations in the parameters of both the LDRW and biophysical models influence the simulated sweat glucose concentrations, providing insights into the extent to which these parameters can be accurately estimated. For the LDRW model, we analyzed three parameters: the ratio of the solute to the volumetric flow ($A$), the mean transit time ($\mu$), and the skewness of the curve ($\lambda$). The biophysical model, being more complex, consisting of 18 parameters. For each parameter in both models, 100 parameter samples were generated using a random number generator that followed a Gaussian distribution. These samples were centered around values sourced from the literature as listed in Table 1, with a standard deviation of 10%. The two models'estimation results were then evaluated to calculate coefficients of variation (CV).

The second analysis aimed at evaluating the relationship between the biophysical and LDRW models, exploring how changes in the biophysical model influenced the derived LDRW model parameters. To clarify, this analysis focused on varying only those biophysical model parameters that exhibited a CV greater than 0.1% in the results of the first sensitivity analysis. Biophysical model parameters with a CV less than 0.1% were deemed to have limited impact on the model's estimation performance and were not considered for further analysis. For each selected biophysical model parameter, 100 samples were generated using a random number generator following a Gaussian distribution centered around referenced literature values listed in Table 1, with a standard deviation of 10%. We then determined the corresponding impulse response of the biophysical model and used the LDRW model to fit these impulse responses. This approach yielded a corresponding distribution of LDRW model parameters, enabling us to assess the interplay between the two models by analyzing the calculated CV.

### Implementation details

The biophysical model simulations were conducted using COMSOL Multiphysics version 6.1. The LDRW model fitting and the inverse estimation by double-loop optimization were implemented in MATLAB R2022a. All simulations and analyses were performed on a personal workstation equipped with an Intel Core i7-12700 CPU, 16 GB RAM, and Windows 11 (64-bit) operating system. No GPU acceleration was used.

## Results

### Estimation performance

**Fig. 5:**
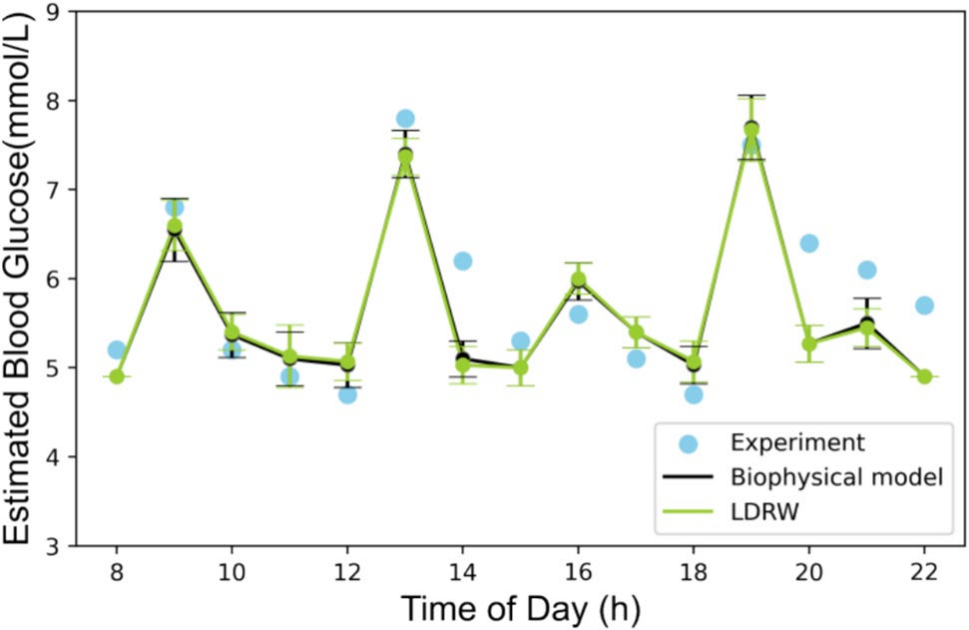
Comparison of experimental blood glucose concentrations with estimates from the biophysical and LDRW models using double-loop optimization. Experimental data come from Lee et al. [40]

The root mean square difference quantifies the difference between the estimated blood glucose concentrations obtained from the biophysical model and those estimated by the LDRW model, amounting to 0.04 $mmol {L}^{-1}$ across all 108 experimental data points. Both models'estimated blood glucose concentrations showed a Pearson correlation coefficient $R$ of 0.98 with the experimental measurements across all data points. The RMSE, MAE, RMSPE, and coefficient of determination further quantify the degree of alignment between the model's estimates and the experimental data, as detailed in Table 3. Shown in Fig. 5 are the estimated blood glucose concentrations from one illustrative experiment, chosen from among the seven experimental datasets considered, obtained by applying the double-loop optimization strategy with both models. A paired two-tailed Wilcoxon signed-rank test was conducted on the estimation results obtained using each model. The result (p = 0.30) indicates that there is no statistically significant difference between the two models in terms of estimation results.

**Table 3:** Comparison of estimation performance metrics for the LDRW and biophysical models

Metrics	LDRW	Biophysical Model
RMSE (mmol L^−1^)	0.85	0.85
MAE (mmol L^−1^)	0.45	0.44
RMSPE (%)	12.41%	12.36%
Pearson correlation coefficient R	0.98	0.98
Coefficient of determination R^2^	0.96	0.96

### Computational efficiency

To compare the computational efficiency of the double-loop optimization strategy when applied to the LDRW and biophysical models, we recorded the computational times required by each model, as shown in Table 4. The biophysical model requires approximately 6 min per data point, whereas the LDRW model requires only 2.6 s, nearly achieving real-time estimation. A paired two-tailed Wilcoxon signed-rank test was conducted on the computational time per data point for each model to assess whether the observed difference in computational efficiency was statistically significant. The result (p = $2.55\times {10}^{-16}$) indicates that the difference is highly significant, confirming that the LDRW model is significantly faster than the biophysical model.

**Table 4:** Comparison of computational time per data point: LDRW vs. biophysical models

Data Sources	Computational Time per Data point for LDRW model (s)	Computational Time Ratio (Biophysical model /LDRW model)
Exp 1[40]	2.3 ± 0.3	151.0
Exp 2[41]	3.8 ± 0.9	128.8
Exp 3[14]	3.1 ± 0.7	140.0
Exp 4[42]	1.8 ± 0.1	162.3
Exp 5[42]	1.9 ± 0.2	173.9
Exp 6[43]	3.6 ± 0.5	127.7
Exp 7[15]	2.0 ± 0.2	149.7
Average	2.6 ± 0.8	151.2 ± 17.0

### Model complexity

The goodness of fit and model complexity of the biophysical model and LDRW model, which were each separately utilized in the double-loop algorithm, were evaluated using AICc. The AICc value for the LDRW model, at 277.3, was lower compared to the biophysical model, which had an AICc value of 313.4.

### Sensitivity analyses

**Fig. 6:**
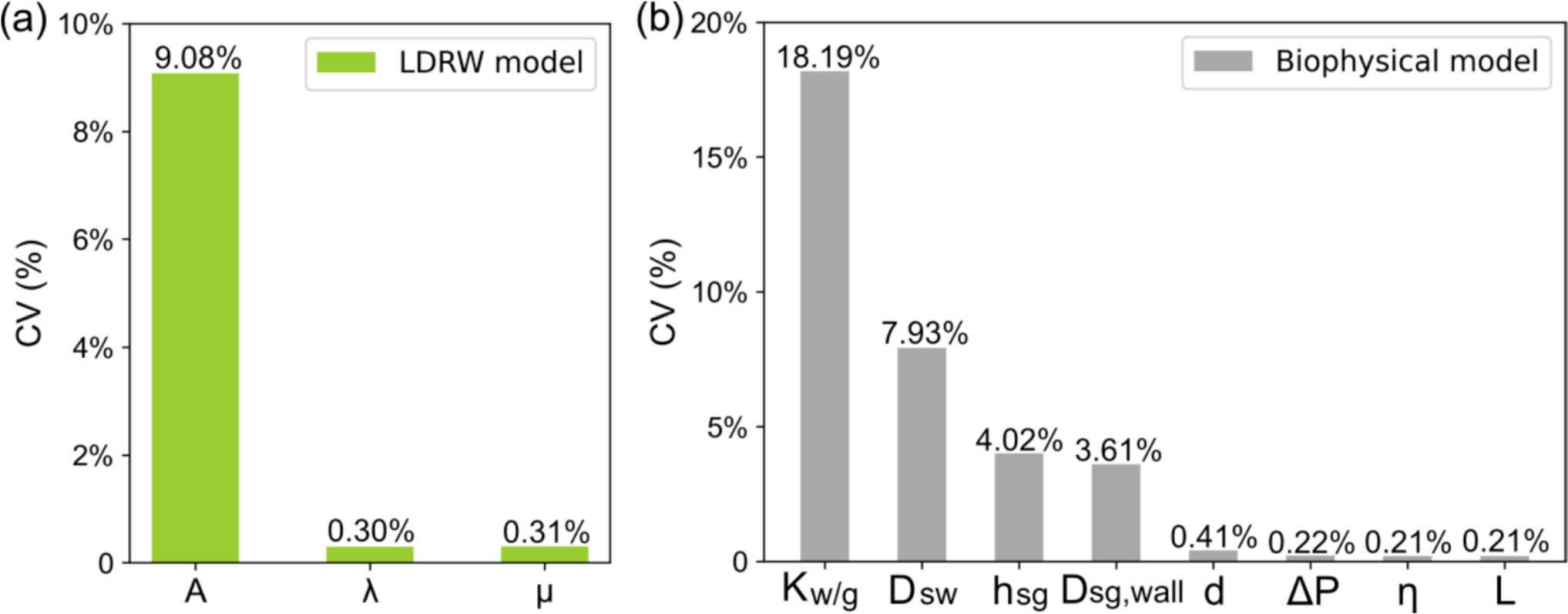
First sensitivity analysis: coefficient of variation (CV) of model parameters for (**a**) LDRW and (**b**) biophysical models

**Fig. 7:**
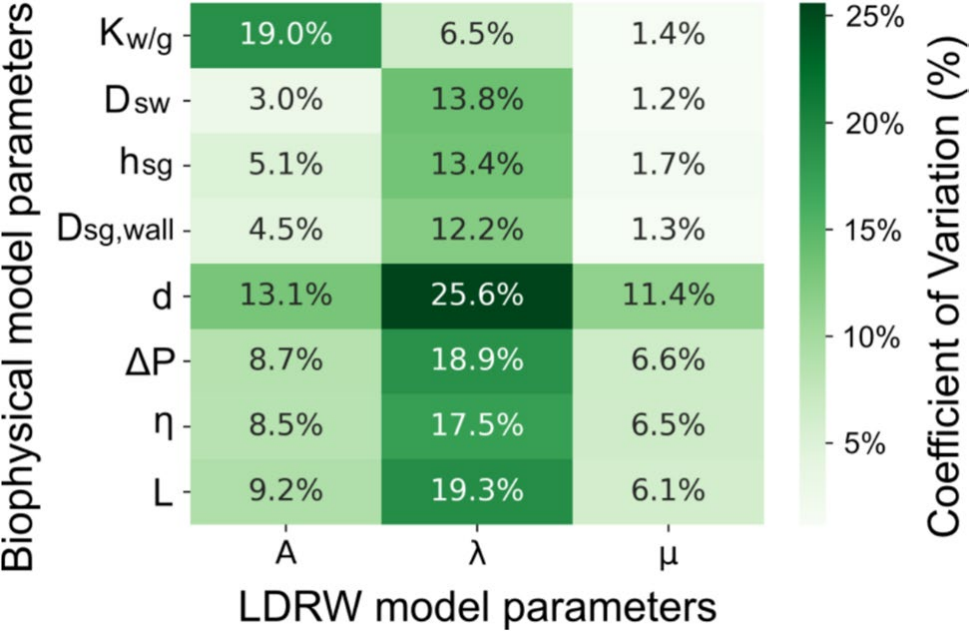
Second sensitivity analysis: coefficients of variation (CV) for LDRW model parameters in response to variations in biophysical model parameters

The first sensitivity analysis was conducted to assess the impact of variations in individual parameters on the accuracy of sweat glucose concentration estimations by the LDRW and biophysical models. In presenting the results in Fig. 6, it should be noted that for the biophysical model, only its parameters exhibiting a CV greater than 0.1% are included. The analysis result indicates that within the LDRW model parameters, the parameter with the largest influence is the ratio of the solute to the volumetric flow ($A$), which showed a CV of 9.08%. In the biophysical model, the ratio of the volumetric flow rate of water to glucose (${K}_{w/g}$) results in the largest CV, equal to 18.19%. The second sensitivity analysis was designed to explore the relationship between variations in individual parameters of the biophysical model and the corresponding changes in the LDRW model parameters. The results are presented in Fig. 7, where a heatmap visualizes the CVs. Within the heatmap, darker shades indicate greater changes in the LDRW parameters resulting from specific variations in the biophysical model parameters.

## Discussion

In this study, we propose substituting our previously proposed biophysical model with the LDRW model to enhance computational-efficiency in estimating blood glucose concentrations from sweat measurements, employing a double-loop optimization strategy.

### Model performance

#### Estimation accuracy

The estimation results obtained from the double-loop optimization strategy using both the LDRW model and the biophysical model show minimal discrepancies, with a low root mean square difference of 0.04 $mmol {L}^{-1}$. Across all 108 data points, the two model's performance in estimating blood concentrations of glucose from sweat is almost identical, as evidenced by an RMSE of 0.85 $mmol {L}^{-1}$ and a Pearson correlation coefficients ($R$) of 0.98. These findings support the replacement of the original biophysical model with the LDRW model, which is specifically designed for diffusion-convection processes. These results demonstrate that the LDRW model retains the estimation accuracy of the biophysical model.

#### Computational efficiency

The use of the LDRW model significantly enhances the computational efficiency of the double-loop optimization. As reported in Table 4, the average computational time per data point for the LDRW model across seven experiments was 2.6 s, compared to 378.9 s for the biophysical model. This substantial reduction in computational time underscores the efficiency of employing the LDRW model within the double-loop optimization strategy. It is worth noting that variations in computational time per data point across different experiments are largely due to differences in experimental conditions, such as the extent of subject's blood glucose concentration fluctuations. For instance, the second experiment (EXP 2 in Table 2), which recorded the longest computational times per data point, exhibited the greatest fluctuations in blood glucose concentrations, with a standard deviation of 5.7 $mmol {L}^{-1}$. Conversely, the forth experiment (EXP 4 in Table 2), with the shortest computational times, had the most stable blood glucose concentrations, indicated by a standard deviation of only 0.1 $mmol {L}^{-1}$. These factors contribute to the observed differences in computational times between these experiments. It is also important to note that in typical glucose monitoring scenarios, glucose measurements are required at relatively low frequencies, typically ranging from 5 to 15 min [47, 48]. The computational speed achieved by the LDRW-based approach ensures timely estimations, making it well-suited for real-time or quasi-real-time glucose monitoring applications.

#### Model complexity

With an AICc value of 277.3, the LDRW model demonstrates a superior balance between model fit quality and model complexity, compared to the biophysical model's higher AICc of 313.4. The lower AICc value indicates that the model is better at explaining the characteristics of the data with fewer parameters. The LDRW model, which utilizes 3 parameters only, achieves performance nearly identical to that of the biophysical model, which uses 18 parameters. This notable reduction in the number of model parameters is crucial, as it not only minimizes the risk of overfitting but also enhances the robustness and reliability of model estimation [49].

### Sensitivity analysis

#### Parameter sensitivity

The results from the first sensitivity analysis demonstrate that parameters from both the LDRW and biophysical models substantially influence the estimation of sweat glucose concentrations. Within the LDRW model, the parameter $A$, representing the ratio of solute to volumetric flow, exhibited a CV of 9.08%, making it the second most influential parameter after the biophysical model's ${K}_{w/g}$. In contrast, the parameter $\lambda$ in the LDRW model, with the smallest CV at 0.30%, ranks higher in sensitivity compared to the fifth most sensitive parameter in the biophysical model, where a total of 18 parameters were assessed but only those with a CV greater than 0.1% are displayed in Fig. 6. This finding underscores a comparable level of sensitivity across the two models.

#### Parameter interpretability

The second sensitivity analysis of the relationship between the parameters of the biophysical and LDRW models supports the possibility of a physiological interpretation of LDRW model parameters. Among all the biophysical model parameters tested, ${K}_{w/g}$ was found to have a most notable influence on the LDRW model parameter $A$, as evidenced by the highest CV of 19.0% for $A$. This correlation indicates a strong dependency of $A$ on variations in ${K}_{w/g}$. This is theoretically expected, given that ${K}_{w/g}$, representing the ratio of the volumetric flow rate of water to the volumetric flow rate of glucose, is conceptually similar to $A$, which represents the ratio of solute (glucose) to volumetric water flow.

Furthermore, changes in the biophysical model's luminal diameter of the sweat gland ($d$) predominantly affect the LDRW model parameter $\mu$. Following $d$, the biophysical model's parameters $\Delta P$, $\eta$, and $L$ exert the next most substantial influence on $\mu$. This is expected, as $\mu$ represents the transit time of glucose, which primarily depends on the velocity of water flow in the sweat gland (${u}_{sg}$). According to Eq. (8), ${u}_{sg}$ is calculated from the ratio of the water flow rate inside the sweat gland (${Q}_{water,sg}$) to the luminal area (${A}_{sg}$), which is directly related to its diameter $d$. Additionally, as per Eq. (6), ${Q}_{water,sg}$ is determined by the pressure difference across the sweat gland ($\Delta P$) and the hydraulic resistance, which correlates with $\eta$ and $L$ as outlined in Eq. (7).

Similarly, $d$ was also found to considerably influence the LDRW model parameter $\lambda$, with $\lambda$ exhibiting the highest CV of 25.6%. Following $d$, $\Delta P$, $\eta$, and $L$ also have the next most substantial impact on $\lambda$. This influence is linked to the definition of the parameter $\lambda$, which represents half of the Peclet number (Pe/2), quantifying the ratio of convective to diffusive transport [17]. The Peclet number is calculated as the product of the compartment length ($L$) and water flow velocity (${u}_{sg}$) divided by the diffusion coefficient. This relationship explains why changes in diffusion coefficients, such as ${D}_{sw}$ and ${D}_{sg,wall}$, notably impact the LDRW model parameter $\lambda$. In addition, in the biophysical model, the glucose uptake by cells in the ISF compartment is represented by the term ${r}_{uptake}$, acting as a sink term in the diffusion-convection Eq. (4). In contrast, the LDRW model simplifies the glucose transport by omitting the sink term. This simplification is supported by our sensitivity analysis, with ${r}_{uptake}$ showing a CV of less than 0.1%, which indicates its negligible influence on the model outputs.

In summary, the parameter values of the LDRW model closely correspond with those of the biophysical model and are amenable to physiological interpretation. This connection and interpretability are crucial for supporting the application of the LDRW model in clinical settings.

### Limitations and future perspectives

The results presented in this paper are based on the limited datasets available in the literature, highlighting the need for future research to incorporate additional data to enhance statistical power. Moreover, the described strategy applies only to biomarkers that rely solely on diffusion-convection transport mechanisms, as exemplified by glucose. Therefore, this method is not directly applicable to biomarkers involving more complex transport mechanisms, such as active transport. This limitation is due to the inherent characteristics of the LDRW model, which is primarily designed to address processes dominated by diffusion and convection. Specific modifications would be necessary to adapt the approach to accommodate these alternative transport mechanisms.

Future research can build upon this work by extending the biophysical model to encompass the transport processes of other biomarkers, such as urea and lactate. Adapting the existing model to these biomarkers would enable the proposed strategy to monitor a broader range of diseases. Such an expansion could significantly enhance the applicability of sweat-sensing technology in disease monitoring, allowing for semi-continuous and non-invasive healthcare solutions.

## Conclusion

In this study, we introduced a computationally efficient method for estimating blood glucose concentrations from sweat measurements, by replacing the previously published 18-parameter biophysical model with a three-parameter LDRW model. Our earlier study demonstrated that the biophysical model significantly outperformed existing state-of-the-art methods. The present results confirm that the LDRW model remains comparable accuracy (R = 0.98) while significantly reducing computational time to only 0.7% of that required by the original method, with an average of 2.6 s per data point. This acceleration brings the method well within the sampling intervals required for current continuous glucose monitoring workflows, enabling semi-continuous blood glucose tracking and proving timely insights for both patients and clinicians. The proposed approach paves the way for prolonged diabetes management through non-invasive sweat-sensing technology.

## References

[1] Lu X et al (2024) Type 2 diabetes mellitus in adults: pathogenesis, prevention and therapy. Sig Transduct Target Ther 9(1):262. 10.1038/s41392-024-01951-9 10.1038/s41392-024-01951-9 PMC11445387 39353925

[2] Magliano D, Boyko EJ (2021) IDF diabetes atlas, 10th edition. Brussels: International Diabetes Federation, 2021. [Online]. Available: https://diabetesatlas.org/resources/previous-editions/. Accessed 3 June 2025

[3] Cole JB, Florez JC (2020) Genetics of diabetes mellitus and diabetes complications. Nat Rev Nephrol 16(7):377–390. 10.1038/s41581-020-0278-5 32398868 10.1038/s41581-020-0278-5 PMC9639302

[4] Siam NH, Snigdha NN, Tabasumma N, Parvin I (2024) Diabetes mellitus and cardiovascular disease: exploring epidemiology, pathophysiology, and treatment strategies. Rev Cardiovasc Med 25(12):436. 10.31083/j.rcm2512436 39742220 10.31083/j.rcm2512436 PMC11683709

[5] Zakir M et al (2023) Cardiovascular Complications of Diabetes: From Microvascular to Macrovascular Pathways. Cureus 15(9):e45835. 10.7759/cureus.45835 37881393 10.7759/cureus.45835 PMC10594042

[6] Gregg EW et al (2024) The burden of diabetes-associated multiple long-term conditions on years of life spent and lost. Nat Med 30(10):2830–2837. 10.1038/s41591-024-03123-2 39090411 10.1038/s41591-024-03123-2 PMC11485235

[7] Templer S, Abdo S, Wong T (2024) Preventing diabetes complications. Intern Med J 54(8):1264–1274. 10.1111/imj.16455 39023283 10.1111/imj.16455

[8] Umpierrez GE et al (2024) Hyperglycemic Crises in Adults With Diabetes: A Consensus Report. Diabetes Care 47(8):1257–1275. 10.2337/dci24-0032 39052901 10.2337/dci24-0032 PMC11272983

[9] Hermanns N, Ehrmann D, Shapira A, Kulzer B, Schmitt A, Laffel L (2022) Coordination of glucose monitoring, self-care behaviour and mental health: achieving precision monitoring in diabetes. Diabetologia 65(11):1883–1894. 10.1007/s00125-022-05685-7 35380233 10.1007/s00125-022-05685-7 PMC9522821

[10] Hoffman MSF, McKeage JW, Xu J, Ruddy BP, Nielsen PMF, Taberner AJ (2023) Minimally invasive capillary blood sampling methods. Expert Rev Med Devices 20(1):5–16. 10.1080/17434440.2023.2170783 36694960 10.1080/17434440.2023.2170783

[11] Lu H, Zada B, Yang L, Dong H (2022) Microneedle-Based Device for Biological Analysis. Front Bioeng Biotechnol 10:851134. 10.3389/fbioe.2022.851134 35528208 10.3389/fbioe.2022.851134 PMC9068878

[12] Ribet F et al (2023) Microneedle Patch for Painless Intradermal Collection of Interstitial Fluid Enabling Multianalyte Measurement of Small Molecules, SARS-CoV-2 Antibodies, and Protein Profiling. Adv Healthcare Mater 12(13):2202564. 10.1002/adhm.202202564 10.1002/adhm.202202564 PMC11468663 36748807

[13] Samant PP et al (2020) Sampling interstitial fluid from human skin using a microneedle patch. Sci Transl Med 12(571):eaaw0285. 10.1126/scitranslmed.aaw0285 33239384 10.1126/scitranslmed.aaw0285 PMC7871333

[14] Moyer J, Wilson D, Finkelshtein I, Wong B, Potts R (2012) Correlation Between Sweat Glucose and Blood Glucose in Subjects with Diabetes. Diabetes Technol Ther 14(5):398–402. 10.1089/dia.2011.0262 22376082 10.1089/dia.2011.0262

[15] Klous L, De Ruiter CJ, Scherrer S, Gerrett N, Daanen HAM (2021) The (in)dependency of blood and sweat sodium, chloride, potassium, ammonia, lactate and glucose concentrations during submaximal exercise. Eur J Appl Physiol 121(3):803–816. 10.1007/s00421-020-04562-8 33355715 10.1007/s00421-020-04562-8 PMC7892530

[16] Yin X et al (2025) “A personalized model and optimization strategy for estimating blood glucose concentrations from sweat measurements,” Computer Methods and Programs in Biomedicine, p. 108743 10.1016/j.cmpb.2025.108743. 10.1016/j.cmpb.2025.108743 40203780

[17] Mischi M, Kalker TA, Korsten EH (2004) Contrast echocardiography for pulmonary blood volume quantification. IEEE Trans Ultrason Ferroelect Freq Contr 51(9):1137–1147. 10.1109/TUFFC.2004.1334846 10.1109/tuffc.2004.1334846 15478975

[18] Sheppard CW, Savage LJ (1951) The random walk problem in relation to the physiology of circulatory mixing. Phys Rev 83:489–490

[19] Turco S et al (2020) Contrast-enhanced ultrasound quantification: from kinetic modeling to machine learning. Ultrasound Med Biol 46(3):518–543. 10.1016/j.ultrasmedbio.2019.11.008 31924424 10.1016/j.ultrasmedbio.2019.11.008

[20] Himeno Y, Ikebuchi M, Maeda A, Noma A, Amano A (2016) Mechanisms underlying the volume regulation of interstitial fluid by capillaries: a simulation study. Integrative Med Res 5(1):11–21. 10.1016/j.imr.2015.12.006 10.1016/j.imr.2015.12.006 PMC5381436 28462092

[21] Ibrahim R, Nitsche JM, Kasting GB (2012) Dermal clearance model for epidermal bioavailability calculations. J Pharm Sci 101(6):2094–2108. 10.1002/jps.23106 22411683 10.1002/jps.23106

[22] Haggerty A, Nirmalan M (2019) Capillary dynamics, interstitial fluid and the lymphatic system. Anaesthesia Intensive Care Med 20(3):182–189. 10.1016/j.mpaic.2019.01.009

[23] Kellen MR, Bassingthwaighte JB (2003) Transient transcapillary exchange of water driven by osmotic forces in the heart. Am J Physiology-Heart Circulatory Physiol 285(3):H1317–H1331. 10.1152/ajpheart.00587.2002 10.1152/ajpheart.00587.2002 PMC3496751 12738617

[24] Longo N, Griffin LD, Langley SD, Elsas LJ (1992) Glucose transport by cultured human fibroblasts: regulation by phorbol esters and insulin. Biochimica et Biophysica Acta (BBA) - Biomembranes 1104(1):24–30. 10.1016/0005-2736(92)90127-8 1550850 10.1016/0005-2736(92)90127-8

[25] Khalil E, Kretsos K, Kasting GB (2006) Glucose Partition Coefficient and Diffusivity in the Lower Skin Layers. Pharm Res 23(6):1227–1234. 10.1007/s11095-006-0141-9 16715366 10.1007/s11095-006-0141-9

[26] Zhang T, Fang HHP (2005) Effective Diffusion Coefficients of Glucose in Artificial Biofilms. Environ Technol 26(2):155–160. 10.1080/09593332608618574 15791796 10.1080/09593332608618574

[27] Sonner Z et al (2015) The microfluidics of the eccrine sweat gland, including biomarker partitioning, transport, and biosensing implications. Biomicrofluidics 9(3):031301. 10.1063/1.4921039 26045728 10.1063/1.4921039 PMC4433483

[28] Schulz IJ (1969) Micropuncture studies of the sweat formation in cystic fibrosis patients. J Clin Invest 48(8):1470–1477. 10.1172/JCI106113 5796359 10.1172/JCI106113 PMC322374

[29] Kestin J, Sokolov M, Wakeham WA (1978) Viscosity of liquid water in the range −8°C to 150°C. J Phys Chem Ref Data 7(3):941–948. 10.1063/1.555581

[30] Wilke K, Martin A, Terstegen L, Biel SS (2007) A short history of sweat gland biology. Intern J of Cosmetic Sci 29(3):169–179. 10.1111/j.1467-2494.2007.00387.x 10.1111/j.1467-2494.2007.00387.x 18489347

[31] Hibbs RG (1958) The fine structure of human eccrine sweat glands. Am J Anat 103(2):201–217. 10.1002/aja.1001030204 13636989 10.1002/aja.1001030204

[32] Nie S, Zhang C, Song J (2018) Thermal management of epidermal electronic devices/skin system considering insensible sweating. Sci Rep 8(1):14121. 10.1038/s41598-018-32152-4 30237407 10.1038/s41598-018-32152-4 PMC6147793

[33] Li C, Wang N, Yao Y, Cui Z, Gao J, Feng D (2022) Density, dynamic viscosity, conductivity and refractive index for mixture D-glucose and deep eutectic solvent (choline chloride + urea) at different temperatures. Phys Chem Liq 60(1):83–94. 10.1080/00319104.2021.1916930

[34] Grussler C, Sepulchre R (2022) Variation diminishing linear time-invariant systems. Automatica 136:109985. 10.1016/j.automatica.2021.109985

[35] Runvik H, Medvedev A (2022) Impulsive time series modeling with application to luteinizing hormone data. Front Endocrinol 13:957993. 10.3389/fendo.2022.957993 10.3389/fendo.2022.957993 PMC9664167 36387902

[36] Cortés JC, Delgadillo‐Alemán SE, Kú‐Carrillo RA, Villanueva RJ (2021) Full probabilistic analysis of random first‐order linear differential equations with Dirac delta impulses appearing in control. *Mathematical Methods in the Applied Sciences* pp.1-20 10.1002/mma.7715.

[37] Mischi M, Kalker T, Korsten E (2003) Videodensitometric Methods for Cardiac Output Measurements. EURASIP J Adv Signal Process 2003(5):862083. 10.1155/S1110865703211185

[38] Danaei G et al (2011) National, regional, and global trends in fasting plasma glucose and diabetes prevalence since 1980: systematic analysis of health examination surveys and epidemiological studies with 370 country-years and 2·7 million participants. The Lancet 378(9785):31–40. 10.1016/S0140-6736(11)60679-X 10.1016/S0140-6736(11)60679-X 21705069

[39] Gill PE, Murray W, Saunders MA (2005) SNOPT: An SQP Algorithm for Large-Scale Constrained Optimization. SIAM Rev 47(1):99–131. 10.1137/S0036144504446096

[40] Lee JW (2010) Fluid and Electrolyte Disturbances in Critically Ill Patients. Electrolyte Blood Press 8(2):72. 10.5049/EBP.2010.8.2.72 21468200 10.5049/EBP.2010.8.2.72 PMC3043756

[41] Boysen TC, Yanagawa S, Sato F, Sato K (1984) A modified anaerobic method of sweat collection. J Appl Physiol 56(5):1302–1307. 10.1152/jappl.1984.56.5.1302 6327585 10.1152/jappl.1984.56.5.1302

[42] La Count TD, Jajack A, Heikenfeld J, Kasting GB (2019) Modeling Glucose Transport From Systemic Circulation to Sweat. J Pharm Sci 108(1):364–371. 10.1016/j.xphs.2018.09.026 30273561 10.1016/j.xphs.2018.09.026

[43] Nyein HYY et al (2019) Regional and correlative sweat analysis using high-throughput microfluidic sensing patches toward decoding sweat. Sci Adv 5(8):eaww9906. 10.1126/sciadv.aaw9906 10.1126/sciadv.aaw9906 PMC6697435 31453333

[44] Chicco D, Warrens MJ, Jurman G (2021) The coefficient of determination R-squared is more informative than SMAPE, MAE, MAPE, MSE and RMSE in regression analysis evaluation. PeerJ Comput Sci 7:e623. 10.7717/peerj-cs.623 34307865 10.7717/peerj-cs.623 PMC8279135

[45] Zhang J, Yang Y, Ding J (2023) Information criteria for model selection. WIREs Comput Stat 15(5):e1607. 10.1002/wics.1607

[46] Dziak JJ, Coffman DL, Lanza ST, Li R, Jermiin LS (2020) Sensitivity and specificity of information criteria. Brief Bioinform 21(2):553–565. 10.1093/bib/bbz016 30895308 10.1093/bib/bbz016 PMC7299313

[47] Hansen KW, Bibby BM (2022) Glycemic Metrics Derived From Intermittently Scanned Continuous Glucose Monitoring. J Diabetes Sci Technol 16(1):113–119. 10.1177/1932296820975822 33269634 10.1177/1932296820975822 PMC8875062

[48] Færch K, Amadid H, Bruhn L, Clemmensen KK, Hulman A, Ried-Larsen M, Blond MB, Jørgensen ME, Vistisen D (2021) Discordance Between Glucose Levels Measured in Interstitial Fluid vs in Venous Plasma After Oral Glucose Administration: A Post-Hoc Analysis From the Randomised Controlled PRE-D Trial. Front Endocrinol 12:753810. 10.3389/fendo.2021.753810 10.3389/fendo.2021.753810 PMC8525890 34675886

[49] Pandey A, Murray RM (2023) Robustness guarantees for structured model reduction of dynamical systems with applications to biomolecular models. Intl J Robust Nonlinear 33(9):5058–5086. 10.1002/rnc.6013

